# Interactions of heparin with key glycoproteins of human respiratory syncytial virus

**DOI:** 10.3389/fmolb.2023.1151174

**Published:** 2023-04-14

**Authors:** Deling Shi, Peng He, Yuefan Song, Robert J. Linhardt, Jonathan S. Dordick, Lianli Chi, Fuming Zhang

**Affiliations:** ^1^ National Glycoengineering Research Center, Shandong University, Qingdao, China; ^2^ Center for Biotechnology and Interdisciplinary Studies, Rensselaer Polytechnic Institute, Troy, NY, United States

**Keywords:** human respiratory syncytial virus, glycoproteins, heparin, pentosan polysulfate, mucopolysaccharide polysulfate

## Abstract

**Introduction:** The unexpected surge of respiratory syncytial virus (RSV) cases following pandemic phase of COVID-19 has drawn much public attention. Drawing on the latest antiviral research, revisiting this heightened annual outbreak of respiratory disease could lead to new treatments. The ability of sulfated polysaccharides to compete for a variety of viruses binding to cell surface heparan sulfate, suggests several drugs that might have therapeutic potential for targeting RSV–glycosaminoglycan interactions.

**Methods:** In the current study, the binding affinity and kinetics of two RSV glycoproteins (RSV-G protein and RSV-F protein) to heparin were investigated by surface plasmon resonance. Furthermore, solution competition studies using heparin oligosaccharides of different lengths indicated that the binding of RSV-G protein to heparin is size-dependent, whereas RSV-F protein did not show any chain length preference.

**Results and discussion:** The two RSV glycoproteins have slightly different preferences for heparin sulfation patterns, but the *N*-sulfo group in heparin was most critical for the binding of heparin to both RSV-G protein and RSV-F protein. Finally, pentosan polysulfate and mucopolysaccharide polysulfate were evaluated for their inhibition of the RSV-G protein and RSV-F protein–heparin interaction, and both highly negative compounds showed strong inhibition.

## 1 Introduction

After 3 years of COVID-19 pandemic, we have faced a triple threat of respiratory illness from severe acute respiratory syndrome coronavirus 2 (SARS-CoV-2), influenza and respiratory syncytial virus (RSV). The data from the United States Centers for Disease Control and Prevention (CDC) shows there is still an increasing number of COVID-19 cases with the emergence of new SARS-CoV-2 variants, and at the same time cases of seasonal flu, and RSV have increased dramatically since the summer of 2022 in the United States. All the three viruses, causing respiratory illnesses, share some common symptoms ranging from mild (such as fever or chills, cough, shortness of breath or difficulty breathing, sore throat, headache, congestion, runny nose, *etc.*) to severe and potentially life-threatening conditions.

RSV was first isolated in 1955 from chimpanzees and later was found in the infants exhibiting severe lower respiratory illness (Blount, Morris, & Savage, 1956; Chanock, Roizman, & Myers, 1957). RSV is a filamentous enveloped, negative-sense, single-stranded RNA virus belonging to the *Orthopneumovirus* genus of the *Pneumoviridae* family (Battles & McLellan, 2019). RSV infection is a global health threat causing substantial illness and mortality among infants and older adults with chronic conditions such as asthma or congestive heart failure (Tong, Amand, Kieffer, & Kyaw, 2020). In the US, RSV results in over 57,000 hospitalizations, 500,000 emergency department visits and 1.5 million outpatient clinic visits among children (<5 years old) (CDC 2019). In addition, in adults there are an estimated 177,000 hospitalizations and 14,000 deaths associated with the RSV infections occurring annually in the US (CDC 2019). RSV infections in children are estimated to annually result in 3.2 million hospitalizations and 94,600 to 149,400 deaths globally (Shi et al., 2017). Currently, there is no widely applied vaccine or medicine to prevent or treat RSV infection. Some vaccines (such as bivalent RSV prefusion F protein–based (RSVpreF) vaccine) are still under development or in clinical trials (Simoes et al., 2022).

The RSV virion surrounds a lipid bilayer displaying the fusion (F), attachment (G) and small hydrophobic (SH) proteins. RSV-G protein and RSV-F protein are the major glycoproteins on the surface of the RSV virion and play critical roles in viral entry (Battles & McLellan, 2019). The RSV-G protein functions primarily to attach virions to target cells by interacting with the molecules on host cell surface. The RSV-F protein primary function is to facilitate fusion of the viral and host cell membranes. Glycosaminoglycans (GAGs), are a class of highly negative charged linear glycans, including heparin/heparan sulfate (HS), chondroitin sulfate (CS)/dermatan sulfate (DS), keratan sulfate (KS), and hyaluronan (HA), commonly found attached on the surface of host cells. These GAGs, especially heparin/HS, play an important role as a co-receptor for many virus-host cell interactions (Aquino & Park, 2016). The G protein containing heparin-binding domain (HBD) has been proved to bind heparin/HS (Krusat & Streckert, 1997; Feldman, Hendry, & Beeler, 1999). Krusat & Streckert (1997) demonstrated that heparin but not HS or CS showed inhibition *in vitro* infection of host cells by RSV, and heparinase digestion of cell surface GAGs reduced the RSV-infection, but not for CS ABC lyase treatment.

The study of GAG–protein interactions at molecular level, an important theme in glycobiology, could result many therapeutic implications. In this work, we analyzed the binding of the two major glycoproteins: RSV-G protein and RSV-F protein with heparin, heparin oligosaccharides of different lengths, and chemically modified heparins using surface plasmon resonance (SPR) to elucidate binding kinetics and structure features (chain size, sulfo group and position) of heparin/HS required for this interaction. Additionally, two highly negative drugs, pentosan polysulfate (PPS) and mucopolysaccharide polysulfate (MPS) were evaluated for their inhibition of the RSV-G protein and RSV-F protein–heparin interaction.

## 2 Materials and methods

### 2.1 Materials

RSV-G protein (Cat: 11070-V08H2), RSV-F protein (Cat: 40628-V08B) were purchased from Sino Biological Inc. The proteins were constructed as follows: 1) a DNA sequence encoding the glycoprotein G extracellular domain (Asn66-Arg297) of human respiratory syncytial virus A (93% homologous with strain rsb1734) (P27022-1) was expressed, with a C-terminal polyhistidine tag; and 2) a DNA sequence encoding the human respiratory syncytial virus fusion (AFX60213.1) (Met1-Ile525) was expressed with a polyhistidine tag at the C-terminus. Unfractionated heparin (15 kDa) was from Celsus Laboratories (Cincinnati, OH). Heparin oligosaccharides from tetrasaccharide (dp4) to octadecasaccharide (dp18) were from Iduron (Manchester, United Kingdom). Desulfated heparins including *N*-desulfated heparin (14 kDa), 2-*O*-desulfated IdoA heparin (13 kDa), 6-*O*-desulfated heparin (13 kDa) were from Iduron (Manchester, United Kingdom). Mucopolysaccharide polysulfate (MPS; 14.5 kDa) was from Luitpold Pharma (Munich, Germany). Pentosan polysulfate (PPS; 6.5 kDa) was from Bene Pharma (Munich, Germany). Sensor streptavidin (SA) chips were from Cytiva (Uppsala, Sweden). SPR experiments were performed using a BIAcore 3000 (Cytiva, Uppsala, Sweden) with Biaevaluation software (version 4.0.1).

### 2.2 Preparation of heparin biochips

Biotinylated heparin was prepared as previously described (Zhang et al., 2022). Heparin (2 mg) and amine-PEG3-Biotin (2 mg) were dissolved in H_2_O (200 µL) added with 10 mg NaCNBH_3_, and reacted at 70°C for 24 h. Then additional NaCNBH_3_ (10 mg) was added and reacted for another 24 h. The biotinylated heparin was desalted and immobilized onto SA chips based on the manufacturer’s protocol. The successful immobilization of heparin was confirmed by the observation of a 200-resonance unit (RU) increase on the sensor chip.

### 2.3 Binding kinetics and affinity measurement

RSV-G protein was diluted in HBS-EP+ buffer (0.01 M 4-(2-hydroxyethyl)-1-piperazineethanesulfonic acid (HEPES), 0.15 M NaCl, 3 mM ethylenediaminetetraacetic acid (EDTA), 0.005% surfactant P20, pH7.4) at concentrations of 500, 250, 125, 62.5, and 31.3 nM, respectively. RSV-F protein was diluted in HBS-EP+ buffer at concentrations of 80, 40, 20, 10, and 5 nM, respectively. Diluted protein samples were injected at a flow rate of 30 μL/min for 3 min at 25°C, followed by dissociation with HBS-EP+ buffer for 3 min. The sensor surface was regenerated by injecting with 30 μL of 0.25% sodium dodecyl sulfonate (SDS) after dissociation time. No protein aggregates were observed in the samples with concentrations ranging from 32 to 500 nM for RSV-G, 5–80 nM for RSV-F before the injection.

### 2.4 Solution competition study between surface-immobilized heparin and heparin oligosaccharides and chemically modified heparins using SPR

Solution competition studies between surface-immobilized heparin and heparin analogs (heparin oligosaccharides and desulfated heparins) in solution were performed as previously described (Shi et al., 2022a). RSV-G protein samples (250 nM) or RSV-F protein samples (40 nM) were pre-mixed with 1,000 nM heparin, heparin oligosaccharides (dp4–dp18) or desulfated heparins, respectively. Then the mixture was injected into the heparin chip at a flow rate of 30 μL/min for 3 min at 25°C. After dissociation, the sensor was regenerated by 30 μL of 0.25% SDS. A control experiment (only protein) was used to test the complete regeneration.

### 2.5 Evaluation of the inhibition activity of PPS and MPS on RSV glycoproteins–heparin interaction using solution competition SPR.

Solution competition studies between surface-immobilized heparin and soluble glycan drugs, PPS and MPS, were performed using SPR to measure their inhibition activity (D. Shi, He, et al., 2022). In brief, RSV-G protein (250 nM) or RSV-F protein (40 nM) pre-mixed with 1,000 nM PPS or MPS were injected at a flow rate of 30 μL/min for 3 min. The resonance signal (RU) decreased when the binding sites on the RSV glycoproteins were occupied by PPS or MPS in solution by preventing RSV glycoprotein binding to the surface-immobilized heparin.

## 3 Results and discussion

### 3.1 Binding affinity and kinetics of glycoprotein–heparin interactions

During pathogen infection, GAGs play an important role in the initial attachment of pathogens to host cells (Kamhi, Joo, Dordick, & Linhardt, 2013). The interaction between heparin/HS and proteins is mainly through the binding of negatively charged groups in the polysaccharide chain to the basic amino acid residues of the proteins (Kjellen & Lindahl, 2018). RSV-G protein and RSV-F protein are glycoproteins on the surface of the RSV virion that facilitate the attachment and fusion of the virion through interactions with host cell surface molecules. Thus, the biochemical and biophysical characterization of the interactions of RSV glycoproteins with host cell GAGs is critically important.

**FIGURE 1:**
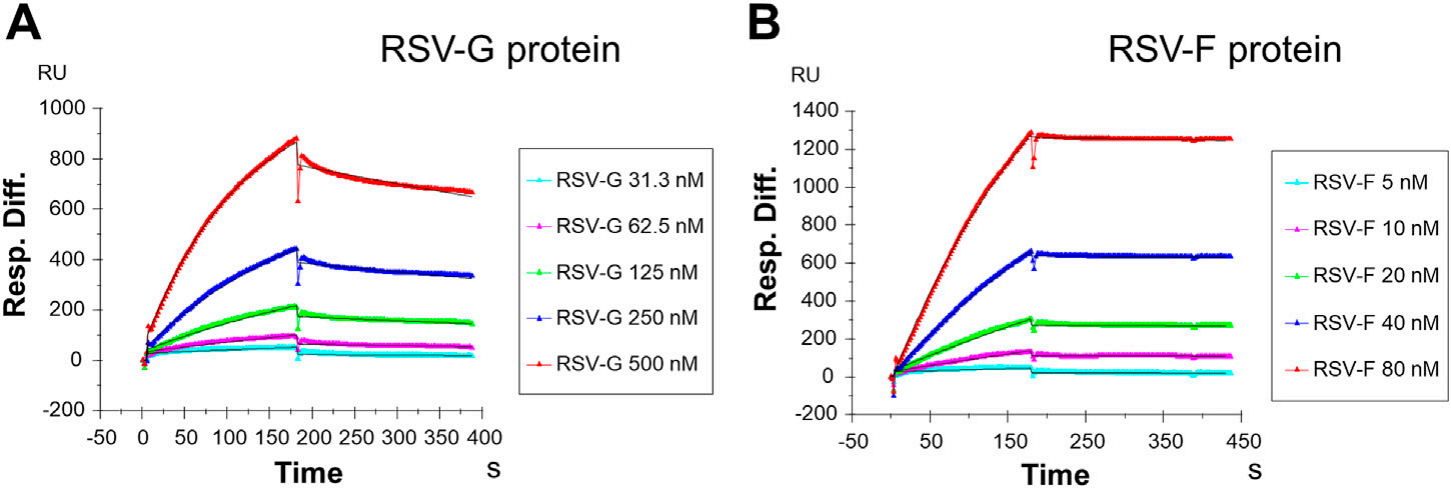
SPR sensorgrams of RSV-G protein and RSV-F protein binding with heparin. **(A)** SPR sensorgrams of RSV-G protein binding with heparin. Concentrations of RSV-G protein (from top to bottom) are 500, 250, 125, 62.5 and 31.3 nM, respectively. **(B)** SPR sensorgrams of RSV-F protein binding with heparin. Concentrations of RSV-F protein (from top to bottom) are 80, 40, 20, 10, and 5 nM, respectively.

In the current study, SPR was used to measure the kinetics and binding affinity of these two RSV glycoproteins interaction with heparin, a highly sulfated analog of host cell HS. Sensorgrams of RSV-G protein or RSV-F protein interactions with immobilized heparin are shown in Figure 1. The sensorgrams were used to determine kinetics (i.e., association rate constant, *k*_*a*_; dissociation rate constant, *k*_*d*_) and affinity (i.e., binding equilibrium dissociation constant, K_D_, where *K_D_* = *k*_*d*_ */k*_*a*_) by globally fitting the sensorgrams using 1: 1 Langmuir binding model (Table 1). Both RSV-G protein and RSV-F protein exhibited an extremely high binding affinity to heparin. The binding affinities were all nanomolar, with 55.5 nM for RSV-G protein and 1.4 nM for RSV-F protein. During chip surface regeneration, the harsh regeneration reagent (0.25% SDS) was used instead of the standard solution (2 M NaCl) to remove heparin-binding proteins.

**TABLE 1:** Summary of kinetic data of RSV-G protein and RSV-F protein binding with heparin.

	*k*_*a*_ (M^−1^s^−1^)	*k*_*d*_ (s^−1^)	*K*_*D*_ (M)
RSV-G protein	1.58 × 10^4^	8.65 × 10^−4^	5.55 × 10^−8^
	(± 245)*	(± 1.08 × 10^−5^)*	(± 6.68 × 10^−9^)**
RSV-F protein	5.26 × 10^4^	5.50 × 10^−5^	1.42 × 10^−9^
	(± 1.07 × 10^−3^)	(± 5.36 × 10^−6^)	(± 3.65 × 10^−10^)*

*The data with (±) in parentheses are the standard deviations (SD) from global fitting of five injections. ** Standard deviation (SD) on triplicated experiments.

### 3.2 Solution competition study on the inhibition activity of heparin oligosaccharides and chemically modified heparins on glycoprotein–heparin interaction

The RSV-G protein is the most variable structural protein among RSV isolates and determines the RSV antigenic groups (RSV A and RSV B) (Sullender, 2000). The variability primarily in the mucin-like domains, and the accumulated are likely to result from selective immune pressure (Pangesti, Abd El Ghany, Walsh, Kesson, & Hill-Cawthorne, 2018). Unlike RSV-G protein, RSV-F protein lacks sequence variation and does not undergo extensive antigenic drift, making it a better therapeutic target. By investigating the molecular properties of RSV glycoprotein–heparin binding, we can work towards suitable drugs that target the interaction and reduce RSV invasion.

**FIGURE 2:**
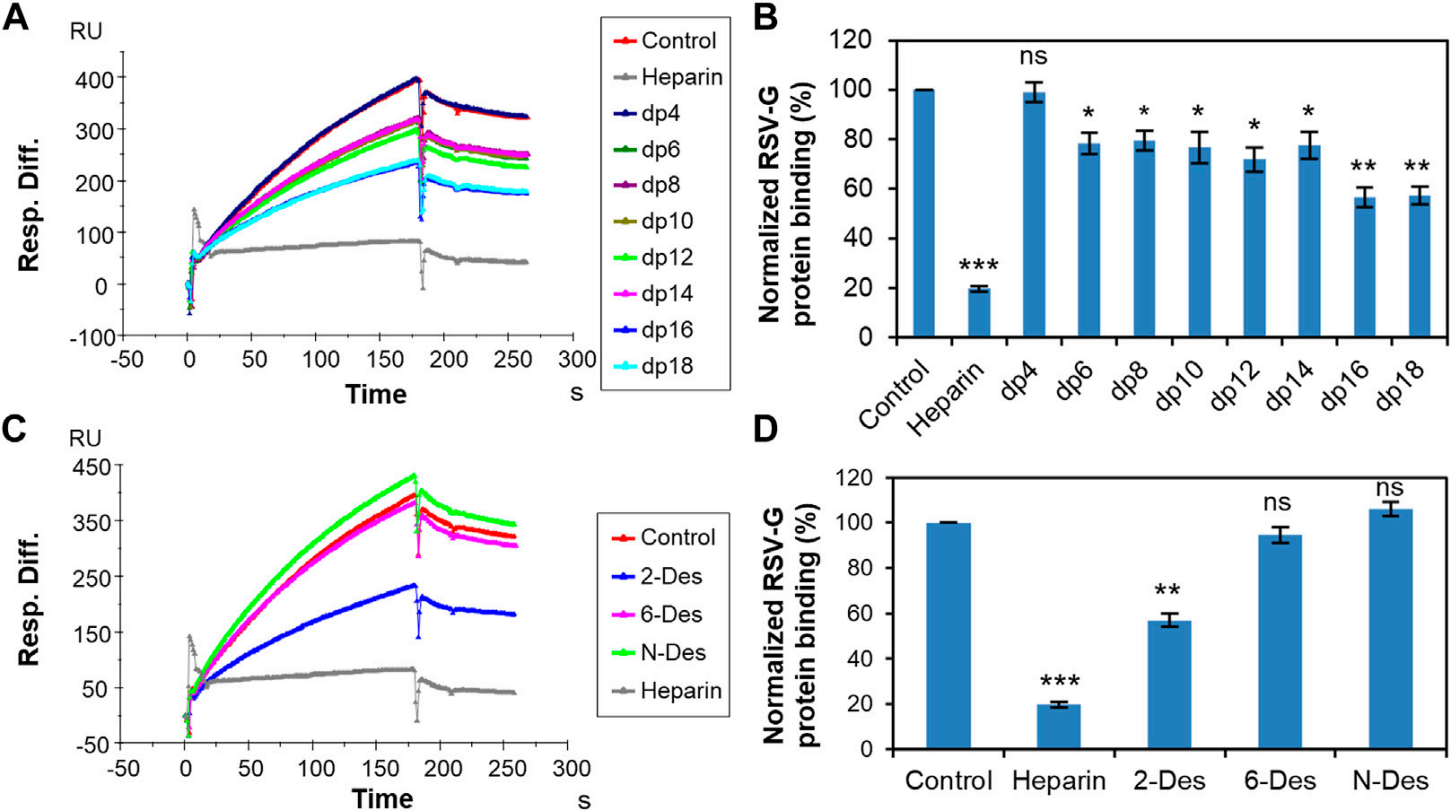
RSV-G protein–heparin interaction inhibited by heparin oligosaccharides or desulfated heparins using solution competition. **(A)** SPR sensorgrams of RSV-G protein–heparin interaction competing with different heparin oligosaccharides. Concentration of RSV-G protein is 250 nM mixed with 1,000 nM of different heparin oligosaccharides. **(B)** Bar graphs (based on triplicate experiments with standard deviation) of normalized RSV-G protein binding to surface-immobilized heparin by competing with different heparin oligosaccharides. **(C)** SPR sensorgrams of RSV-G protein–heparin interaction competing with different desulfated heparins. Concentration of RSV-G protein is 250 nM mixed with 1,000 nM of different desulfated heparins. **(D)** Bar graphs (based on triplicate experiments with standard deviation) of normalized RSV-G protein binding to surface-immobilized heparin by competing with different desulfated heparins. Statistical analysis was performed using unpaired two-tailed *t*-test (ns: *p* > 0.05 compared to the control, *: *p* ≤ 0.05 compared to the control, **: *p* ≤ 0.01 compared to the control, ***: *p* ≤ 0.001 compared to the control).

The negatively charged regions of cell surface HS are considered to be the attachment point of the RSV and binding is primarily mediated by positively charged amino acids located between the two mucin-like domains of RSV-G protein (Feldman et al., 1999; Hallak, Collins, Knudson, & Peeples, 2000; Battles & McLellan, 2019). Hallak, Spillmann, Collins, & Peeples (2000) found that *N*-sulfation but not 6-*O*-sulfation or 2-*O*-sulfation is important for RSV infection and dp10 is the minimum size that can neutralize RSV infectivity using an improved recombinant green fluorescent protein-expressing RSV to assay infection. Here, solution competition experiments were performed to examine the effect of the saccharide chain length and sulfation pattern of heparin on the interaction of heparin and RSV-G protein. Different heparin oligosaccharides (dp4-dp18) or chemically desulfated heparins at 1,000 nM concentration were applied in the competition analysis (Figure 2). The signal decreased meaning the binding sites on the RSV-G protein were occupied by solution heparin instead of the surface-immobilized heparin. Interestingly, 6-*O*-desulfated heparin and *N*-desulfated heparin could barely inhibit the binding of RSV-G protein and immobilized heparin. (Figures 2C, D). In contrast to Hallak’s results, we found both *N*-sulfation and 6-*O*-sulfation of heparin are particularly important in heparin–RSV-G protein interaction, and high levels of sulfation are required for binding. The inhibition was size-dependent with the minimal heparin length to effectively inhibit the RSV-G protein–heparin interaction being dp6 (Figures 2A, B). It should be noted that the inhibition of the interaction is significantly enhanced when the heparin chain length ≥ dp16. The binding of RSV-G protein and heparin showed a preference for longer saccharide chains.

**FIGURE 3:**
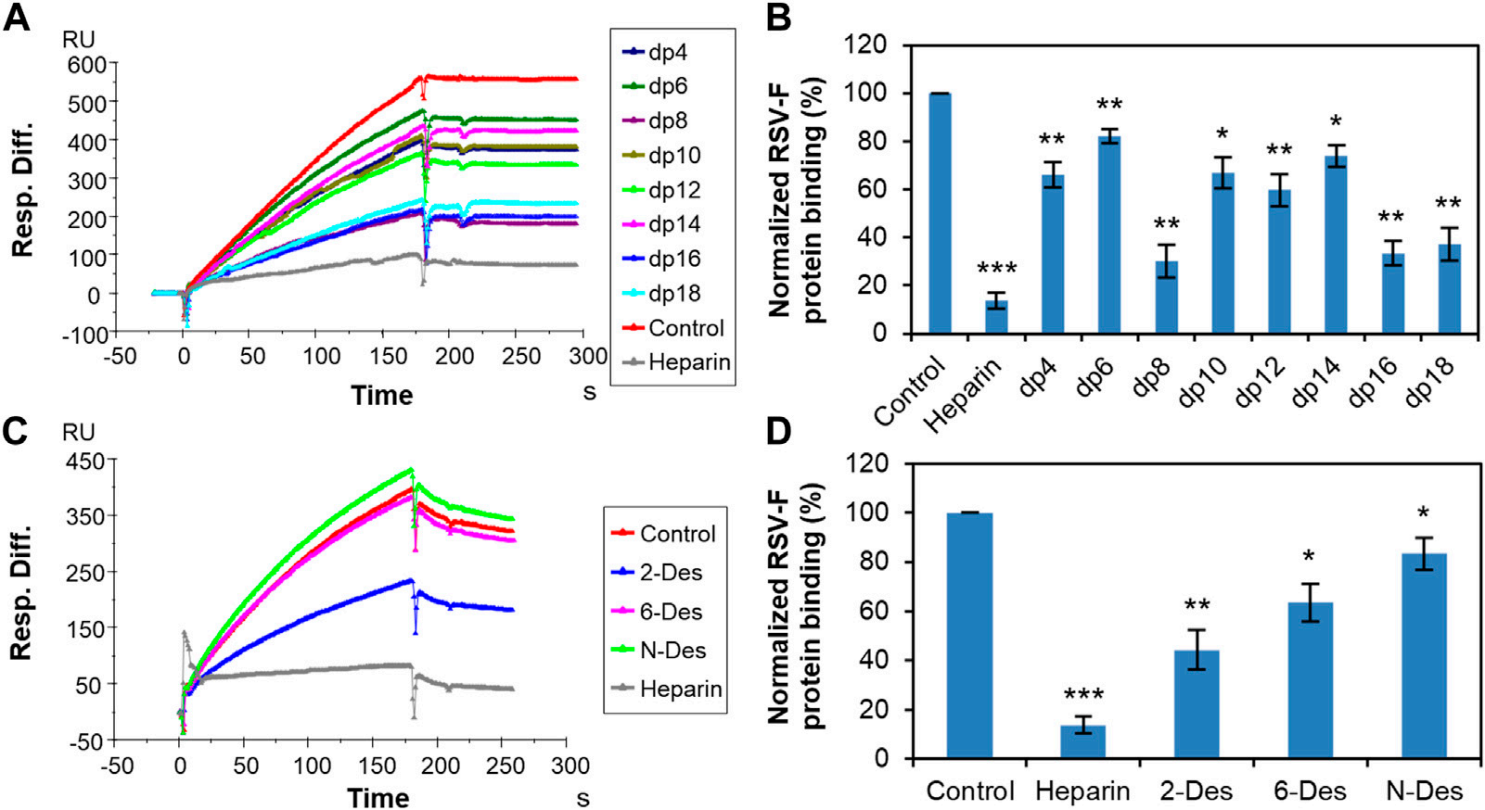
RSV-F protein–heparin interaction inhibited by heparin oligosaccharides or desulfated heparins using solution competition. **(A)** SPR sensorgrams of RSV-F protein–heparin interaction competing with different heparin oligosaccharides. Concentration of RSV-F protein is 40 nM mixed with 1,000 nM of different heparin oligosaccharides. **(B)** Bar graphs (based on triplicate experiments with standard deviation) of normalized RSV-F protein binding to surface-immobilized heparin by competing with different heparin oligosaccharides. **(C)** SPR sensorgrams of RSV-F protein–heparin interaction competing with different desulfated heparins. Concentration of RSV-F protein is 40 nM mixed with 1,000 nM of different desulfated heparins. **(D)** Bar graphs (based on triplicate experiments with standard deviation) of normalized RSV-F protein binding to surface-immobilized heparin by competing with different desulfated heparins. Statistical analysis was performed using unpaired two-tailed *t*-test (*: *p* ≤ 0.05 compared to the control, **: *p* ≤ 0.01 compared to the control, ***: *p* ≤ 0.001 compared to the control).

In the case of RSV-F protein, previous studies demonstrated that this protein independently interacts with heparin/HS and facilitates virus attachment and infectivity (Feldman, Audet, & Beeler, 2000). Solution competition SPR experiments were next performed to examine the structural characteristics of the interaction between RSV-F protein and heparin (Figure 3). Interestingly, inhibition of RSV-F protein–heparin interaction appears to be independent of heparin length, with dp8 inhibiting binding as effectively as dp18 (Figures 3A, B). Nevertheless, chains longer than dp16 showed more efficient inhibition. We then investigated the ability of chemically desulfated heparins to inhibit the interaction of RSV-F protein with surface-immobilized heparin (Figures 3C, D). The results showed that the binding is strong charge-dependent and shows preference for the *N*-sulfation and 6-*O*-sulfation of heparin, with 2-*O*-sulfation exerting a weaker inhibitory effect. This suggests that the binding of RSV-F protein to heparin is more selective for a specific structure than that of RSV-G protein.

### 3.3 Potential Anti-RSV activity of PPS and MPS

**FIGURE 4:**
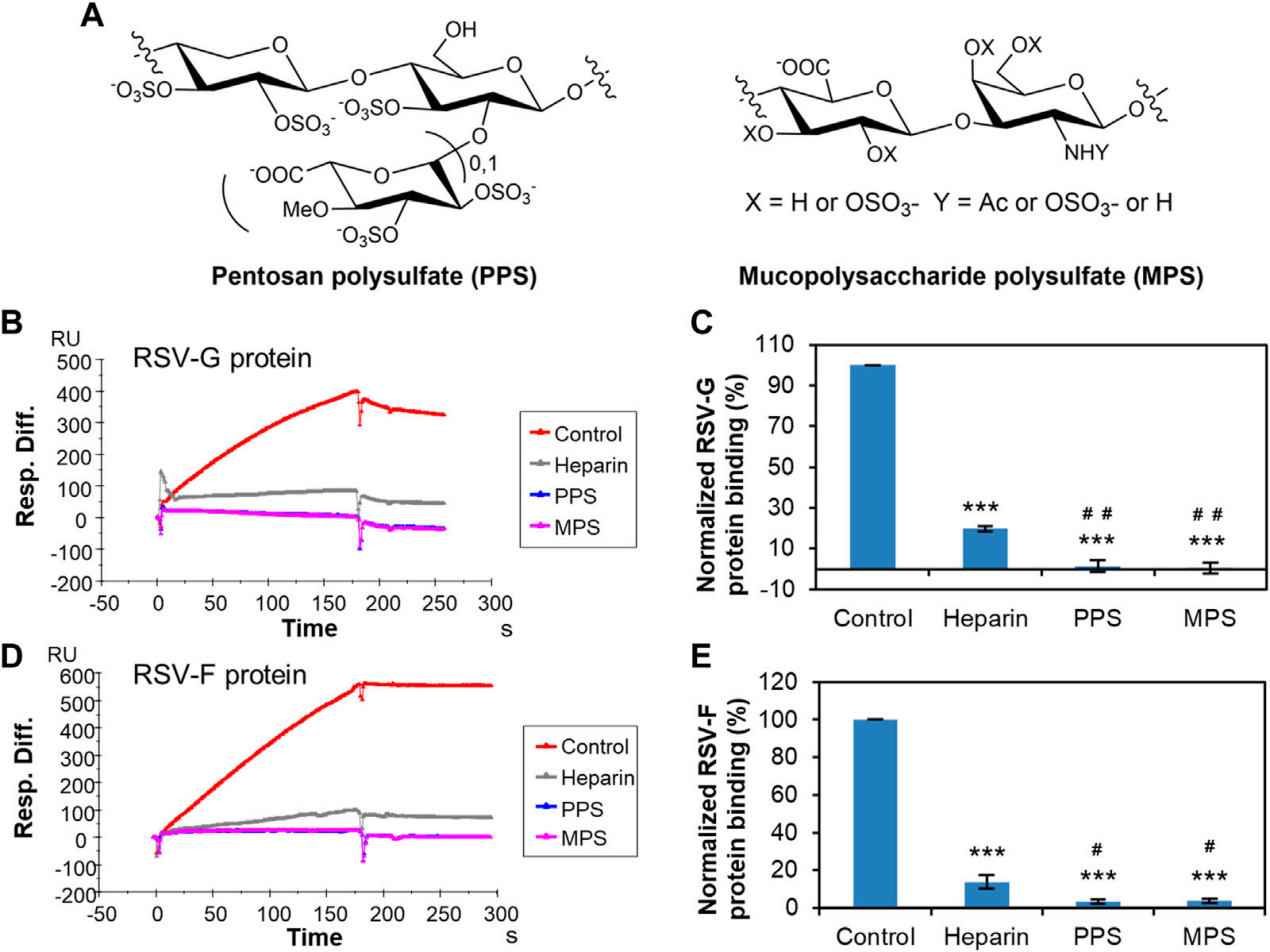
Solution competition between heparin and PPS or MPS. **(A)** Structure of PPS and MPS. **(B)** SPR sensorgrams of RSV-G protein–heparin interaction competing with PPS or MPS. Concentration of RSV-G protein is 250 nM mixed with 1,000 nM of PPS or MPS. **(C)** Bar graphs (based on triplicate experiments with standard deviation) of normalized RSV-G protein binding to surface-immobilized heparin by competing with PPS or MPS. **(D)** SPR sensorgrams of RSV-F protein–heparin interaction competing with PPS or MPS. Concentration of RSV-F protein is 40 nM mixed with 1,000 nM of PPS or MPS. **(E)** Bar graphs (based on triplicate experiments with standard deviation) of normalized RSV-F protein binding to surface-immobilized heparin by competing with PPS or MPS. Statistical analysis was performed using unpaired two-tailed *t*-test (***: *p* ≤ 0.001 compared to the control, #: *p* ≤ 0.05 compared to heparin, ##: *p* ≤ 0.01 compared to heparin).

PPS is a semi-synthetic sulfated polysaccharide, that is, chemically and structurally similar to heparin (Frileux, 1951), and its structure is shown in Figure 4A. The average PPS disaccharide contains >3 sulfo groups. PPS (Elmiron^®^) is currently the only United States Food and Drug Administration (FDA)-approved oral therapy for the relief of bladder pain or discomfort associated with interstitial cystitis. The safety and efficacy of PPS have been demonstrated in multiple open-label and comparative clinical trials in different populations (Anderson & Perry, 2006). PPS has showed strong activity to inhibit infection of SARS-CoV-2 (Ennemoser et al., 2021; Bertini et al., 2022; Zhang et al., 2022), Monkeypox virus (Shi et al., 2022b), and human immunodeficiency virus (HIV-I) (Srivastava, Sekaly, & Chiasson, 1993). MPS, another heparin analogue, is derived from mammalian cartilage (Lane, Michalski, Van Ross, & Kakkar, 1977). MPS is highly sulfated with more than 4 sulfo groups per disaccharide unit (Figure 4A) and mimics many of the properties of heparin. Creams containing MPS are widely used to treat eczema and have been proven to be effective and safe (Li, Li, Xiang, & Li, 2021). Our previous studies also demonstrated that MPS inhibit the interaction of heparin/HS and virus, such as SARS-CoV-2 (Zhang et al., 2022) and Monkeypox virus (Shi et al., 2022b). Furthermore, both PPS and MPS exhibit reduced anticoagulant potential (Lane et al., 1977; Bertini et al., 2022), making these excellent potential candidates as antiviral agents. Based on these previous studies, PPS and MPS were selected for further evaluation as potential therapeutic or prophylactic agents against RSV.

Solution competition showed that both PPS and MPS showed significant inhibition of the binding of surface-immobilized heparin to RSV-G protein or RSV-F protein. PPS and MPS potently inhibited the RSV-G protein–heparin interaction by 98.6% ± 2.9% and 99.5% ± 2.6% (Figures 4B, C), respectively, while inhibiting the RSV-F protein–heparin interaction by 96.5% ± 1.2% and 96.2% ± 1.3% (Figures 4D, E), respectively. These inhibitory effects were even significantly higher than that of heparin in solution (80.3% ± 1.1% for RSV-G protein and 86.3% ± 2.5% for RSV-F protein, respectively). Here, the slight difference in the inhibition efficacy of PPS and MPS on RSV-G and RSV-F proteins also reflect the bias of the two proteins for binding to sulfated polysaccharides. RSV-G protein is more likely to bind polysaccharides with stronger charges, while RSV-F protein has a slight structural bias in addition to preferring high charge. Since highly negatively charged PPS and MPS exhibit anti-RSV activity, these are promising drugs for preventing the invasion and infection of RSV by inhibiting the interaction between RSV and HS on the cell surface. As anti-RSV drug candidates, the mechanism of PPS or MPS against RSV warrants further investigation. Due to the different molecular weights and sulfation degrees of PPS and MPS, more studies are needed to compare the inhibitory activity and mechanism of these two molecules on viral infection.

## 4 Conclusion

Investigating the molecular properties of RSV glycoprotein–heparin interaction, we can develop drugs that target the interaction and reduce RSV invasion. SPR analysis revealed that the binding affinity (*K_D_*) of RSV-G protein and RSV-F protein were all at nanomolar concentrations, and the affinity of RSV-F protein was even stronger. Solution competition studies indicated that these two RSV glycoproteins exhibit different preferences for heparin chain lengths and sulfation patterns. Competition assays demonstrated that efficient binding of RSV-G protein requires longer saccharide chain, which is not necessary for RSV-F protein. We also found that 6-*O*-desulfated heparin and *N*-desulfated heparin could barely inhibit the binding of RSV-G protein to surface-immobilized heparin, whereas all chemically modified heparin derivatives showed reduced resonance signal in competition analysis of RSV-F protein–heparin interaction with the inhibition efficacy lower than that of heparin. Thus, all the sulfation sites are important for interaction between the RSV-G protein or RSV-F protein and heparin, although *N*-sulfo and 6-*O*-sulfo groups are much more critical for the binding to RSV-G protein. Most importantly, highly negatively charged PPS and MPS show promise as therapeutic and/or preventative antiviral drugs against RSV. It is promising to develop PPS or MPS related drugs targeting the interaction between RSV and HS on the cell surface to inhibit the invasion and infection of RSV.

## Data Availability

The original contributions presented in the study are included in the article/Supplementary Material, further inquiries can be directed to the corresponding authors.
